# Repeatability of Phoneme-Guided Versus Conventional Acquisition of Smiling and Rest-Related Facial States Using 3D Stereophotogrammetry: A Within-Participant Study

**DOI:** 10.3390/bioengineering13090994

**Published:** 2026-08-27

**Authors:** Weijing Dang, Liang Lyu, Yong Shen, Ji Wang, Yi Yu, Qirui Wang, Mingjin Zhang, Jiajia Zheng, Dawei Liu

**Affiliations:** 1 Department of Orthodontics, Peking University School and Hospital of Stomatology and National Center of Stomatology, Beijing 100081, China; dangweijing0320@163.com (W.D.); wangji@pkuss.bjmu.edu.cn (J.W.); yuyinaive@outlook.com (Y.Y.); wqrdent@163.com (Q.W.); 1610303110@pku.edu.cn (M.Z.); 2 National Clinical Research Center for Oral Diseases and National Engineering Research Center of Oral Biomaterials and Digital Medical Devices, Beijing 100081, China; sy1281408675@163.com (Y.S.); zhengjiajia@pkuss.bjmu.edu.cn (J.Z.); 3 Beijing Key Laboratory of Digital Stomatology, Beijing 100081, China; 4 Department of Nursing, Peking University School and Hospital of Stomatology and National Center of Stomatology, Beijing 100081, China; 5 Eighth Clinical Division (Shijingshan Clinical Division), Peking University School and Hospital of Stomatology and National Center of Stomatology, Beijing 100043, China

**Keywords:** three-dimensional facial imaging, stereophotogrammetry, phoneme-guided acquisition, acquisition standardization, repeatability, digital orthodontics

## Abstract

Repeatable acquisition of smiling and rest-related facial states is essential for longitudinal three-dimensional (3D) facial imaging, yet the effect of task instructions may depend on the state and measurement. This within-participant study compared conventional (CONV) and state-specific phoneme-guided (PHON) acquisition in 162 adults. For SMILE, CONV elicited a posed smile, whereas PHON used /tʃ/ articulation followed by maintenance of the resulting perioral configuration; for the REST-related condition, CONV elicited relaxed rest, whereas PHON used sustained /m/ to define a closed-lip reference configuration rather than unconstrained physiological rest. Each protocol–state condition was recorded twice. Within-session repeatability was characterized by absolute error between repeated acquisitions, technical error of measurement, intraclass correlation coefficient [ICC(2,1)], and Bland–Altman agreement. State-specific protocol differences were assessed using paired Wilcoxon signed-rank tests with Holm correction. During SMILE, PHON reduced median error for the 3D Sn–Gn distance (3.79 vs. 2.36 mm), interlabial distance (3.48 vs. 1.84 mm), and sagittal lip step (1.33 vs. 0.66 mm; adjusted *p* < 0.001), but increased commissure-distance error (1.50 vs. 1.89 mm; adjusted *p* = 0.007). During the REST-related condition, no prespecified linear outcome differed after correction. Phoneme guidance changed, rather than uniformly improved, repeatability, and its effect depended on facial state and outcome.

## 1. Introduction

Three-dimensional (3D) facial surface imaging is increasingly combined with intraoral scanning and cone-beam computed tomography to create virtual patients for diagnosis, simulation, prosthetic and orthodontic planning, and longitudinal outcome assessment [1,2,3]. Contemporary stereophotogrammetric, structured-light, smartphone, and other optical systems can provide high technical performance under controlled conditions [4,5,6,7,8]. Technical performance alone, however, does not establish the repeatability of a live-subject record.

Repeatability of sequential facial records is also affected by head positioning, involuntary movement, execution of the requested facial state, surface reconstruction, and landmark identification. Natural head position can be reproduced under standardized conditions [9], but live-subject error is spatially heterogeneous and is often greatest around mobile facial structures [7,10]. Acquisition instructions are therefore part of the measurement system rather than a neutral prelude to image capture.

Smiling and resting facial states are routinely documented in dentofacial records, yet neither is mechanically fixed. Repeated 3D recordings have demonstrated variability in elicited expressions [11,12], resting lip position [13], smile tasks [14], and other nonverbal movements [15]. Dynamic studies further indicate that verbal and nonverbal facial gestures can differ in reproducibility [16,17]. SMILE and REST consequently present different standardization challenges: smiling requires repeatable activation of a coordinated perioral pattern, whereas resting requires suppression of speech, swallowing, lip adjustment, and other low-amplitude movements.

A phoneme cue may provide a more explicit motor target than an abstract request to “smile” or “relax.” Smile-related soft-tissue dimensions remain relevant to dentofacial assessment [18], and phonetic tasks have long been used to elicit mandibular and perioral configurations [19,20,21,22]. Nevertheless, a phoneme-derived pose cannot be assumed to reproduce unconstrained physiologic rest or a conventional posed smile. Evidence remains limited on whether state-specific phoneme cues alter end-to-end repeatability across both facial states and whether any effect is consistent across measurement outcomes.

Although the technical performance of 3D facial imaging systems is well established, existing evidence does not show whether phoneme-based motor cues alter end-to-end, within-session repeatability across facial states and clinically relevant outcomes. This gap limits evidence-based selection of acquisition instructions for clinical and longitudinal workflows.

The primary objective was to compare conventional and state-specific phoneme-guided acquisition instructions for SMILE and a REST-related condition in a within-participant repeated-acquisition design. We evaluated immediate within-session repeatability for nine SMILE outcomes and six REST-related outcomes. Landmark- and region-level displacement, state-by-protocol contrasts, and stable-landmark acquisition-quality indicators were secondary analyses. We hypothesized that phoneme guidance would have state- and outcome-specific effects rather than uniformly reduced error across the facial surface.

## 2. Materials and Methods

### 2.1. Study Design and Ethics

This single-center, within-participant method-comparison study evaluated repeated 3D facial acquisitions under two instruction protocols (conventional and phoneme-guided) and two facial conditions (SMILE and REST-related). The study was conducted in accordance with the Declaration of Helsinki and approved by the Institutional Review Board of Peking University School and Hospital of Stomatology (protocol code PKUSSIRB-2025108043; approved on 8 February 2025). Written informed consent was obtained from all participants. The study compared acquisition instructions and did not assign a therapeutic intervention.

### 2.2. Participants and Sample-Size Considerations

Participants were recruited consecutively from the Department of Orthodontics, Peking University School and Hospital of Stomatology, between December 2025 and April 2026. Eligible participants were adults aged 18 years or older who could understand and perform the acquisition tasks. Exclusion criteria were an evident facial soft-tissue abnormality, previous maxillofacial trauma or surgery, a clinically apparent neuromuscular disorder, or a craniofacial anomaly likely to compromise standardized surface capture.

An a priori planning benchmark was derived using a conservative independent-groups normal approximation (expected difference, 3.0 mm; pooled standard deviation, 5.57 mm; two-sided α = 0.05; power, 90%), yielding approximately 73 participants per group. Because the final design was within-participant, this calculation served only as a minimum recruitment benchmark rather than endpoint-specific power assurance. Consecutive recruitment yielded 162 complete participants, each of whom contributed both protocols.

### 2.3. Three-Dimensional Facial Acquisition System

Facial surfaces were acquired with a 3dMDface stereophotogrammetry system (3dMD, Atlanta, GA, USA) after manufacturer-recommended calibration. Participants were seated upright in natural head position, established by self-balancing and visual fixation. The forehead and both ears were exposed; glasses and facial accessories were removed. Ambient lighting and the participant-to-camera configuration were held constant across repeated acquisitions. All scans were obtained by one trained orthodontic nurse. Each surface was inspected immediately for gross motion artifact or incomplete coverage and was reacquired when either problem was visually evident. Rejected or reacquired attempts were not prospectively logged, so condition-specific reacquisition rates could not be reconstructed. The analysis therefore concerns accepted scan pairs entering the measurement workflow [4,6,7,8,9,23].

### 2.4. Conventional and Phoneme-Guided Acquisition Tasks

Each participant completed conventional (CONV) and phoneme-guided (PHON) acquisitions in both facial conditions. For SMILE-CONV, participants adopted and held a posed smile without speaking. For SMILE-PHON, participants briefly articulated /tʃ/ and maintained the resulting perioral configuration during capture. For REST-CONV, participants relaxed the facial musculature and maintained a resting facial pose. For REST-PHON, participants gently sustained the bilabial nasal /m/ and maintained the resulting closed-lip configuration during capture. These phoneme-guided tasks were defined motor targets; neither was assumed to be interchangeable with spontaneous smiling or unconstrained physiological rest. Operational English translations of the standardized verbal instructions are provided in Table S1.

The starting instruction protocol (CONV or PHON) was randomized at acquisition. However, the realized participant-specific order of the four protocol–state conditions was not retained in the analysis dataset; sequence balance and carryover effects therefore could not be reconstructed. Within each condition, replicate order was fixed (R1 followed by R2). The operator initiated capture after visually confirming that the requested configuration was stable. Cue-to-capture latency, task-hold duration, intervals between repeated acquisitions, and intervals between protocol–state conditions were not instrumented or prospectively recorded, and no formal washout or neutral-reset interval was prescribed.

### 2.5. Landmark Identification and Coordinate System

A calibrated examiner manually identified 20 soft-tissue landmarks on every accepted facial surface using Geomagic Studio 2013 (3D Systems, Rock Hill, SC, USA). Formal blinding to protocol and replicate was not implemented because task-related facial configurations and structured scan identifiers could reveal these conditions. No independent repeated-landmarking subset was available to estimate intra-examiner reliability. Landmark selection and nomenclature followed established 3D facial anthropometric practice [24,25,26]. The exported coordinate convention was X = participant’s right, Y = superior, and Z = anterior. Landmark definitions and facial-region assignments are provided in Table S2. For each landmark, displacement between R1 and R2 was calculated as the Euclidean 3D distance. Consequently, the reported R1–R2 differences include both acquisition variability and manual landmark-placement variability.

### 2.6. Derived Measures and Analysis Dataset

Nine predefined outcomes were evaluated for SMILE and six shared outcomes for REST (Table S3). Euclidean 3D distances were calculated for EnR–EnL (intercanthal distance), AlR–AlL (nasal width), Sn–Gn, Stmi–Gn, Stms–Stmi (interlabial distance), ChR–ChL (commissure distance), and GoR–GoL (bigonial distance), as applicable to the state-specific outcome set. Two sagittal components were calculated as absolute differences between the corresponding Z coordinates: |ZSn − ZLi| and |ZLs − ZLi|. The terminology distinguishes 3D Euclidean distances from single-axis components. For each participant, protocol, state, and outcome, the signed difference was R1 minus R2 and the absolute error was |R1 − R2|.

The analysis contained one row per participant–protocol–state outcome combination. Protocol, state, and replicate were parsed from structured scan identifiers, which uniquely encoded these fields and served as the canonical analysis key. Completeness was verified against the expected 1296 acquisitions (162 participants × 2 protocols × 2 states × 2 replicates).

**Figure 1:**
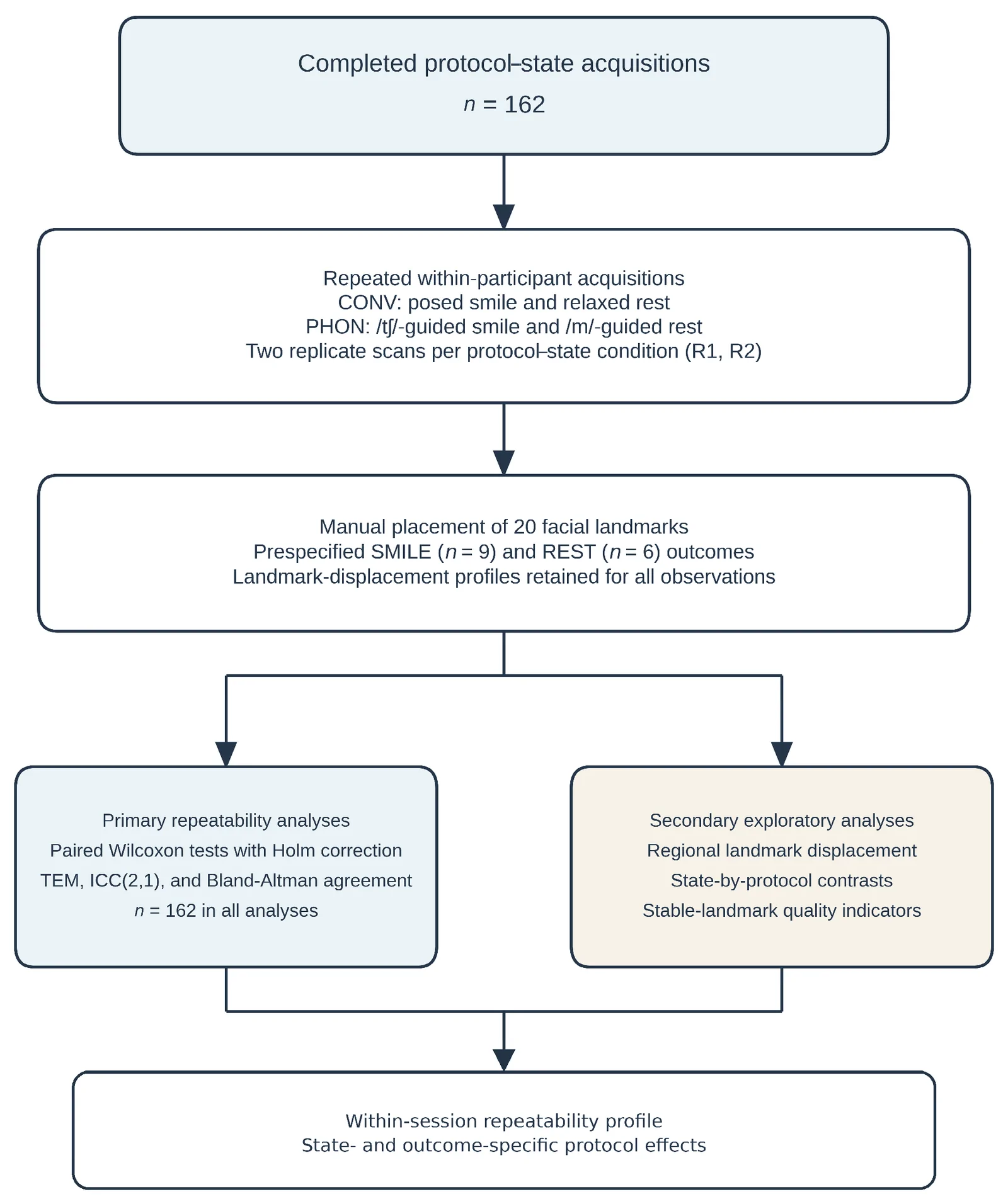
Repeated-acquisition and analysis workflow. Participants completed all protocol–state acquisitions (*n* = 162). CONV denotes conventional posed-smile and relaxed-rest instructions; PHON denotes /tʃ/-guided smile and /m/-guided closed-lip reference acquisition. The starting instruction protocol was randomized, although the realized participant-specific sequence was not retained. Each protocol–state condition was recorded twice in fixed replicate order (R1 followed by R2), followed by manual placement of 20 facial landmarks and derivation of predefined SMILE and REST-related outcomes. Primary analyses comprised paired Wilcoxon tests with Holm correction, TEM, ICC(2,1), and Bland–Altman agreement; secondary analyses comprised regional landmark displacement, state-by-protocol contrasts, and stable-landmark acquisition-quality indicators. All analyses retained all 162 participants. TEM, technical error of measurement; ICC, intraclass correlation coefficient.

**Figure 2:**
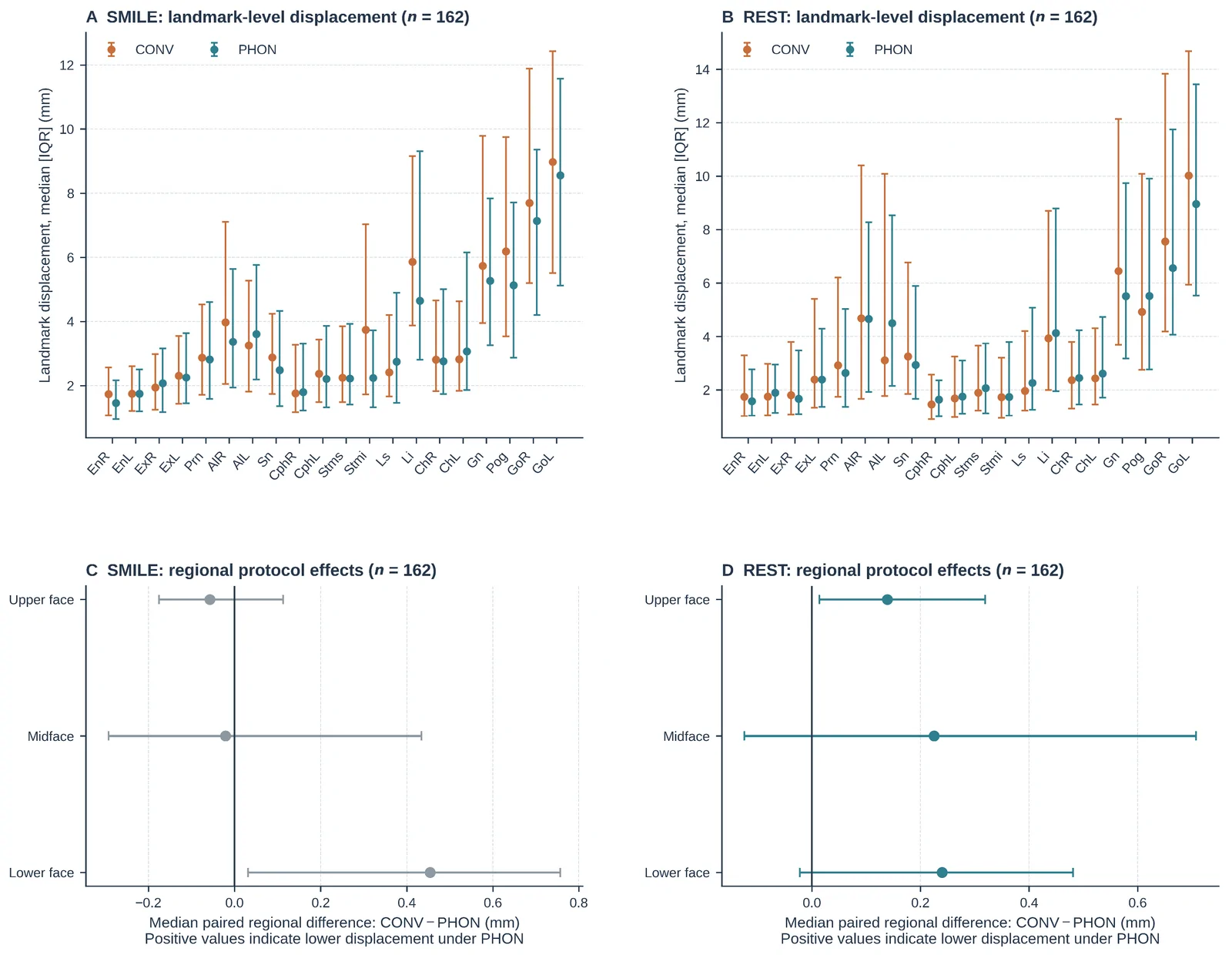
State-specific landmark- and region-level end-to-end displacement in the complete cohort (*n* = 162). (**A**,**B**) Median R1−R2 Euclidean displacement for each landmark under CONV and PHON; error bars show the interquartile range. (**C**,**D**) Median participant-level protocol difference in mean regional displacement (CONV − PHON); bars show percentile-bootstrap 95% confidence intervals. Positive values indicate lower displacement under PHON; adjusted *p*-values are reported in Table S4. Displacement includes acquisition variability and manual landmark-placement variability.

In practical terms, each participant completed four task conditions (SMILE-CONV, SMILE-PHON, REST-CONV, and REST-PHON), with two accepted 3D scans per condition. Twenty landmarks spanning the eye corners, nose, lips, chin, and mandibular angles were placed on each surface (Table S2). Distances derived from these landmarks were compared between R1 and R2; smaller absolute R1–R2 differences indicated better repeatability. Figure 1 summarizes the experimental sequence, and Figure 2 provides landmark- and region-level visual summaries.

### 2.7. Secondary Acquisition-Quality Indicators

To characterize acquisition behavior without changing the analysis population, we calculated a stable-landmark displacement index for each participant, protocol, and facial state as the median R1–R2 Euclidean displacement across seven relatively stable landmarks (EnR, EnL, ExR, ExL, Prn, AlR, and AlL). A state-specific upper fence (Q3 + 1.5 × IQR) was derived from the pooled protocol-level index distribution [27] and used only as an exploratory flag for unusually high displacement. Neither the continuous index nor the flag was used to exclude scans or participants; all 162 participants remained in every inferential analysis.

### 2.8. Statistical Analysis

Reliability and agreement were reported with reference to the Guidelines for Reporting Reliability and Agreement Studies (GRRAS) [28]. All 162 participants were retained in every inferential analysis and the nine SMILE outcomes and six REST outcomes were treated as prespecified state-specific comparison families. SMILE and REST were analyzed separately because they used different motor tasks and outcome sets. Absolute errors were summarized by the median, interquartile range (IQR), mean, and 90th percentile (P90). Within each state, CONV and PHON absolute errors were compared using two-sided paired Wilcoxon signed-rank tests. Family-wise error was controlled using Holm correction across the nine SMILE outcomes and separately across the six REST outcomes; adjusted *p* < 0.05 was considered statistically significant.

The participant-level effect estimate was the median paired difference in absolute error (CONV minus PHON), with a percentile-bootstrap 95% confidence interval based on 10,000 resamples. Positive values indicate lower error under PHON. Paired rank–biserial correlation (r) was reported as a standardized effect size. For the six outcomes shared by SMILE and REST, a secondary state-by-protocol analysis compared the participant-level protocol effect between states; Holm correction was applied across the six contrasts. Because the bootstrap interval for the median and the Wilcoxon signed-rank test summarize different distributional features, they were interpreted as complementary rather than interchangeable statistics.

Within each protocol–state condition, the technical error of measurement (TEM) was calculated as TEM = √[Σ(Di^2^)/(2N)], where Di is the signed R1–R2 difference [29]. Intraclass correlation coefficients were estimated as ICC(2,1), a two-way random-effects, absolute-agreement, single-measure model, with 95% confidence intervals [30,31]. Agreement was additionally summarized using Bland–Altman bias and 95% limits of agreement [32]. Because ICC depends on between-participant variability, it was interpreted together with absolute error and TEM. Landmark-region comparisons were secondary and Holm-adjusted within state. The continuous stable-landmark displacement index was compared between protocols using paired Wilcoxon tests, and upper-fence flags were compared using exact McNemar tests; both sets of state-specific *p*-values were Holm-adjusted across SMILE and REST.

In this study, repeatability denotes agreement between repeated acquisitions obtained with the same device, operator, session, and protocol. Reproducibility is reserved for agreement under changed measurement conditions or for independent regeneration of the analysis, whereas accuracy denotes closeness to a reference value. The study was designed to estimate repeatability, not scanner accuracy.

Analyses were performed in Python 3.13.5 (Python Software Foundation, Wilmington, DE, USA) using NumPy 2.3.5, pandas 2.2.3, SciPy 1.17.0, Matplotlib 3.10.8, and Pillow 12.2.0. Deterministic seeds were used for bootstrap resampling and figure jitter.

The acquisition and analysis workflow is shown in Figure 1.

## 3. Results

### 3.1. Participant Characteristics and Analysis Completeness

All 162 participants completed both instruction protocols, both facial states, and both repeated acquisitions. The dataset therefore contained 1296 accepted scans with no metric-level missingness. Mean age was 31.9 years (standard deviation, 5.9; range, 20–49 years), and 114 participants (70.4%) were female. Every inferential analysis used the same 162 participants (Table 1).

### 3.2. State-Specific Landmark and Regional Error Patterns

Landmark-level displacement was lowest around the orbital landmarks and generally higher at perioral and mandibular landmarks in both states (Figure 2A,B). Although the individual-landmark profiles appeared broadly similar between protocols, derived measurements combine displacement at more than one landmark and can therefore show protocol effects not evident in marginal landmark summaries. During SMILE, none of the three regional comparisons remained significant after Holm correction; the largest median CONV − PHON difference was 0.45 mm for the lower face (adjusted *p* = 0.072). During REST, median differences were 0.14, 0.23, and 0.24 mm for the upper face, midface, and lower face, respectively; each paired rank test yielded adjusted *p* = 0.014 (Figure 2C,D; Table S4). Bootstrap confidence intervals excluded zero for the upper face but crossed zero for the midface and lower face, indicating uncertainty in the median effect estimates despite the rank-based distributional differences.

### 3.3. Primary State-Specific Protocol Comparisons

**Figure 3:**
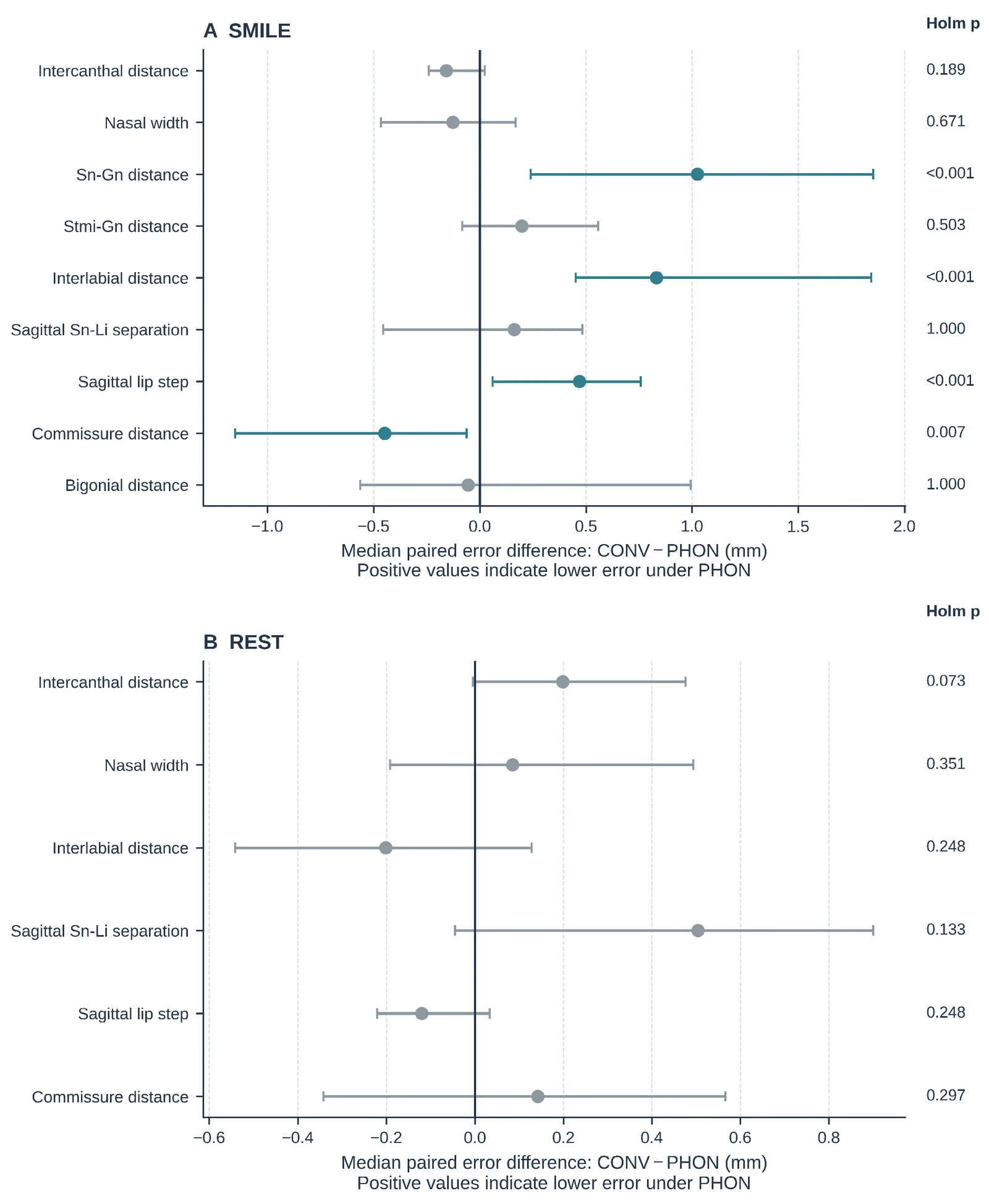
State-specific effects of phoneme-guided versus conventional acquisition instructions in the complete cohort (*n* = 162). Points represent the median participant-level difference in absolute error (CONV − PHON); bars show percentile-bootstrap 95% confidence intervals. Positive values favor PHON. *p*-values are two-sided paired Wilcoxon signed-rank tests with Holm correction within each state.

State-specific protocol comparisons and complete descriptive distributions are summarized in Table 2 and Table S7, and Figure 3 and Figure S1. During SMILE, PHON reduced median absolute error for the 3D Sn–Gn distance from 3.79 to 2.36 mm (median paired difference, 1.03 mm; rank–biserial r = 0.41), for 3D interlabial distance from 3.48 to 1.84 mm (0.83 mm; r = 0.45), and for absolute sagittal lip step from 1.33 to 0.66 mm (0.47 mm; r = 0.44); Holm-adjusted *p* < 0.001 for all three. The significant median paired reductions therefore ranged from 0.47 to 1.03 mm. In contrast, 3D commissure-distance error was greater under PHON (1.50 vs. 1.89 mm; median paired difference, −0.45 mm; r = −0.29; adjusted *p* = 0.007). The other five SMILE outcomes did not differ after correction.

During the REST-related condition, none of the six predefined linear outcomes differed after Holm correction. The smallest unadjusted *p*-value was observed for 3D intercanthal distance (median error, 1.12 mm under CONV vs. 0.85 mm under PHON; adjusted *p* = 0.073). Thus, the primary-outcome-level analyses did not support a uniform repeatability benefit for the /m/-guided closed-lip task.

### 3.4. State-by-Protocol Contrasts

**Figure 4:**
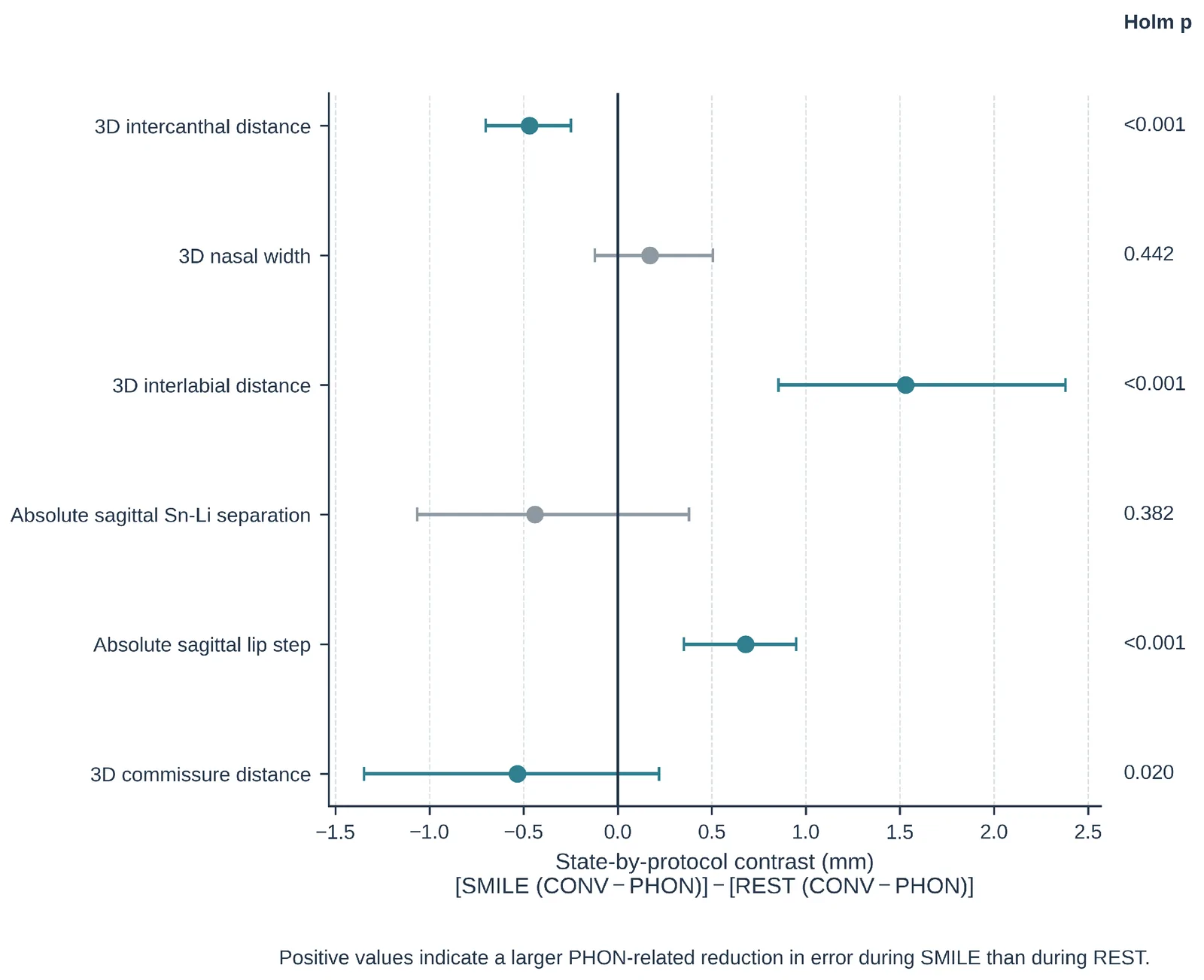
State dependence of the acquisition-instruction effect in the complete cohort (*n* = 162). Points show the median state-by-protocol contrast, defined as [SMILE (CONV − PHON)] − [REST (CONV − PHON)]; bars show percentile-bootstrap 95% confidence intervals. *p*-values are two-sided Wilcoxon signed-rank tests with Holm correction across the six shared measures.

For the six outcomes shared by SMILE and REST, Holm-adjusted state-by-protocol contrasts were significant for 3D intercanthal distance, 3D interlabial distance, absolute sagittal lip step, and 3D commissure distance (Table 3; Figure 4). The largest positive contrast was observed for 3D interlabial distance (1.53 mm; 95% confidence interval, 0.86–2.38 mm; adjusted *p* < 0.001), indicating a larger PHON-related reduction in error during SMILE than during REST. Negative contrasts for intercanthal and commissure distances indicated that the protocol effect differed in the opposite direction between states. These interaction estimates do not imply a significant protocol effect within either state unless supported by the corresponding state-specific analysis.

### 3.5. TEM, ICC, Agreement, and Secondary Acquisition-Quality Indicators

Within-protocol repeatability estimates also varied by state and outcome (Table S5). During SMILE, PHON lowered TEM for the 3D Sn–Gn distance (from 5.28 to 2.84 mm), 3D interlabial distance (from 5.95 to 3.56 mm), and sagittal lip step (from 2.63 to 1.29 mm), whereas commissure-distance TEM increased from 2.31 to 5.46 mm. During REST, PHON lowered TEM and increased ICC for intercanthal distance, nasal width, and commissure distance, but did not improve every outcome. Bland–Altman analyses likewise showed outcome-specific changes in bias and limits of agreement (Table S5; Figure S3). Complete commissure-distance error distributions are shown in Figure S4.

All repeatability and acquisition-quality analyses retained *n* = 162. The continuous stable-landmark displacement index did not differ after Holm correction during SMILE (CONV median, 2.16 mm; PHON median, 2.16 mm; adjusted *p* = 0.882) or REST (1.91 vs. 2.09 mm; adjusted *p* = 0.051). Upper-fence high-displacement flags occurred in 14 CONV and 10 PHON SMILE pairs (adjusted exact McNemar *p* = 0.481) and in 34 CONV and 27 PHON REST pairs (adjusted *p* = 0.031) (Table S6; Figure S2). Because the upper fences were study-specific and data-derived, and because the continuous REST index did not reach the corrected significance threshold, the flag-based REST result was treated as exploratory.

## 4. Discussion

This study evaluated phoneme guidance as an acquisition-stage standardization strategy for SMILE and a REST-related condition. The /tʃ/-guided SMILE task reduced within-session error for selected lower-face outcomes but increased commissure-distance error, whereas the /m/-guided closed-lip task did not improve any predefined linear outcome after multiplicity correction. Regional displacement and the data-derived high-displacement flag produced secondary REST-related signals, but the continuous stable-landmark index did not differ after correction. Overall, phoneme guidance changed repeatability in a state- and outcome-specific manner rather than providing a uniform improvement.

These findings reinforce the distinction between scanner performance and live-subject workflow performance. Comparative evaluations of 3D facial scanners can demonstrate high technical capability under controlled conditions [4,5,6,7,8,23], whereas live-subject studies identify additional variability around mobile facial regions [7,10]. The end-to-end errors reported here incorporate task execution, image acquisition, surface processing, and manual landmark placement; they should not be interpreted as camera accuracy or isolated neuromuscular variability.

For SMILE, the observed pattern is consistent with evidence that posed smiles and other nonverbal expressions are difficult to reproduce and that verbal facial gestures impose different motor constraints [11,12,14,15,16,17]. The /tʃ/-guided task reduced error for Sn–Gn distance, interlabial distance, and sagittal lip step, but not for transverse commissure distance. The broadly similar individual-landmark profiles in Figure 2A,B are not inconsistent with these derived measurement effects, because interlandmark distances depend on the combined movement of their component landmarks. One possible explanation is that the cue constrained selected sagittal and predominantly vertical components while permitting heterogeneous lateral excursion; muscle activity and mandibular motion were not measured, so this explanation remains hypothetical. The resulting pose should therefore be regarded as a distinct standardized task configuration rather than a globally better or more natural smile.

The statistically significant median paired reductions ranged from 0.47 to 1.03 mm. The study did not prespecify a minimal clinically important difference, and no universal threshold can be inferred from these data. These magnitudes are similar to the order of manual 3D soft-tissue landmark-placement error reported in prior studies [24,25]. Their practical importance therefore depends on the intended application and the magnitude of change being monitored; statistical significance alone should not be interpreted as universal clinical benefit. The simultaneous increase in commissure-distance error under PHON further supports an outcome-specific interpretation.

REST requires a separate interpretation. Resting lip position varies within and between sessions [13], and phonetic tasks can alter mandibular and perioral posture [19,20,21,22]. Sustained /m/ defined an active closed-lip reference configuration and did not improve the six predefined REST linear outcomes after Holm correction; it therefore cannot be considered a generally superior resting instruction. Small regional differences and fewer upper-fence flags suggest that an explicit closed-lip target may influence aspects of the acquisition workflow not captured by the selected linear outcomes. However, the continuous stable-landmark index was not significant after correction and the upper fence was data-derived; the threshold-based result is exploratory and requires external validation.

The selected phonemes were chosen as pragmatic motor-task anchors rather than universal representations of smile or rest. The brief /tʃ/ cue provided a discrete transition to a holdable perioral endpoint, whereas /m/ provided a simple sustained bilabial-closure target. Other phonetic options were not systematically compared in this study. In other linguistic settings, functionally equivalent cues should be selected by matching the intended articulatory endpoint rather than literal spelling; candidate cues should be easy to understand and perform, minimally affected by coarticulation, and locally validated for the intended facial outcome before clinical use.

From an engineering perspective, replacing an abstract instruction with a speech-related cue did not have a constant effect across facial states or outcomes. Acquisition protocols should therefore define the target state, verbal cue, capture timing, repeated-acquisition procedure, and outcome set explicitly. Conventional and phoneme-guided tasks should not be interchanged within a longitudinal series without validation, because task-specific gains in one outcome may coexist with deterioration in another. This measurement-specific interpretation is consistent with recent validation studies that evaluate facial-scanning technologies and processing workflows for defined clinical outputs rather than assume interchangeability across systems or tasks [23,33,34].

Downstream registration could reduce residual pose and correspondence error: rigid iterative closest point (ICP) alignment can address head-position mismatch, whereas nonrigid ICP variants or spline-based deformation models can improve local surface correspondence. Because flexible registration may also attenuate the true task-related soft-tissue deformation of interest, these algorithms require task-specific validation and were not introduced post hoc into the present landmark-based analysis.

Strengths of this study include the complete within-participant dataset, retention of all 162 participants in every analysis, repeated acquisitions for four protocol–state conditions, state-specific multiplicity control, direct paired effect estimates, complementary TEM/ICC/Bland–Altman reporting, and secondary quality indicators that did not determine inclusion.

Several procedural limitations affect causal interpretation and full-workflow replicability. Although the starting instruction protocol was randomized, the realized participant-specific sequence was not retained; sequence balance could not be verified and order effects could not be modeled. R1 always preceded R2. Familiarization or learning could reduce later variability, whereas fatigue, expression drift, or carryover could increase it; the direction and magnitude of any order effect are therefore indeterminate. Timing variables were not instrumented; no formal washout or neutral-reset interval was prescribed, and rejected or reacquired attempts were not logged. The study evaluated static held configurations rather than dynamic phoneme-to-pose kinematics. Consequently, the estimates apply to accepted scans and may overstate the repeatability and efficiency of the complete acquisition workflow.

External validity is limited by the single-center setting, one scanner, one acquisition operator, and one manual landmark examiner. Formal blinding and an independent repeated-landmarking study were not performed; effects of approximately 0.5–1.0 mm may therefore overlap manual landmark-placement variability. The sample was 70.4% female, and sex-stratified analyses were not prespecified, limiting generalizability to more balanced populations. Because the sample-size calculation was a non-endpoint-specific recruitment benchmark, some individual outcomes may remain underpowered and nonsignificant findings should not be interpreted as equivalence. Repeats were consecutive and within session, so the findings cannot be extrapolated to monitoring over days, weeks, or months. Several outcomes showed long-tailed or heteroscedastic differences, so fixed Bland–Altman limits of agreement should be interpreted descriptively. Naturalness, comfort, execution time, muscle activity, and clinical acceptability were not measured.

The structured landmark and scan-level data could support future machine-learning research on automated facial-state classification and acquisition-quality flagging. Future prospective work will extend this framework by using hardware-synchronized high-frame-rate capture to characterize phoneme-to-pose kinematics; evaluating rigid and nonrigid registration methods; prospectively logging initial-capture failures, reacquisition time, and task timing; and separating acquisition variability from landmarking variability through blinded repeated landmarking on the same surfaces. Non-identifiable anatomical schematics and task-state exemplars, together with multicenter validation, will further improve engineering interpretability and clinical translation.

## 5. Conclusions

Phoneme guidance changed immediate within-session repeatability in an outcome-specific manner. The /tʃ/-guided SMILE task reduced error for selected lower-face outcomes but increased commissure-distance error, whereas sustained /m/ did not improve the predefined linear outcomes in the REST-related closed-lip condition after multiplicity correction. These phoneme-derived configurations should be treated as defined acquisition reference tasks rather than interchangeable substitutes for unconstrained natural smile or physiological rest. The significant reductions were submillimeter to approximately 1 mm and should be judged against application-specific tolerances. Because all repeats were consecutive and within-session, the findings cannot be extrapolated to longitudinal monitoring; cross-session, multicenter, multi-operator, and language-specific validation is required before clinical implementation.

## Figures and Tables

**Table 1:** Participant characteristics and analysis completeness.

Characteristic	Value
Participants, *n*	162
Age, mean ± SD (range), years	31.9 ± 5.9 (20–49)
Sex, *n* (%)	Female 114 (70.4%); Male 48 (29.6%)
Expected and analyzed acquisitions, *n*	1296 and 1296
Complete SMILE paired participants, *n* (%)	162 (100.0%)
Complete REST paired participants, *n* (%)	162 (100.0%)
Participants retained in every inferential analysis, *n* (%)	162 (100.0%)
Metric-level missing observations, *n*	0

SD, standard deviation. All inferential analyses retained all 162 participants.

**Table 2:** State-specific paired comparisons of absolute R1–R2 error in the complete cohort (*n* = 162).

State	Measurement	CONV Median [IQR], mm	PHON Median [IQR], mm	CONV − PHON Median Difference [95% CI], mm	Rank–Biserial r	Holm-Adjusted *p*
SMILE	3D intercanthal distance	0.76 [0.31, 1.37]	0.96 [0.45, 1.75]	−0.16 [−0.24, 0.02]	−0.19	0.189
	3D nasal width	1.37 [0.55, 2.12]	1.36 [0.69, 2.35]	−0.13 [−0.47, 0.17]	−0.11	0.671
	3D Sn-Gn distance	3.79 [1.34, 6.52]	2.36 [1.03, 4.06]	1.03 [0.24, 1.85]	0.41	<0.001
	3D Stmi-Gn distance	1.56 [0.82, 2.89]	1.38 [0.70, 2.42]	0.20 [−0.08, 0.56]	0.14	0.503
	3D interlabial distance	3.48 [1.29, 9.41]	1.84 [0.71, 4.02]	0.83 [0.45, 1.84]	0.45	<0.001
	Absolute sagittal Sn-Li separation	2.25 [0.97, 4.42]	2.16 [1.01, 4.84]	0.16 [−0.45, 0.48]	0.01	1.000
	Absolute sagittal lip step	1.33 [0.41, 3.54]	0.66 [0.30, 1.70]	0.47 [0.06, 0.76]	0.44	<0.001
	3D commissure distance	1.50 [0.68, 3.02]	1.89 [0.92, 4.15]	−0.45 [−1.15, −0.06]	−0.29	0.007
	3D bigonial distance	3.70 [1.68, 6.81]	3.69 [1.80, 6.19]	−0.06 [−0.56, 0.99]	0.01	1.000
REST	3D intercanthal distance	1.12 [0.52, 2.10]	0.85 [0.34, 1.55]	0.20 [0.00, 0.48]	0.23	0.073
	3D nasal width	1.29 [0.60, 2.75]	1.41 [0.55, 2.44]	0.08 [−0.19, 0.49]	0.08	0.351
	3D interlabial distance	1.21 [0.46, 2.72]	1.37 [0.64, 3.06]	−0.20 [−0.54, 0.13]	−0.17	0.248
	Absolute sagittal Sn-Li separation	2.71 [1.18, 4.33]	1.99 [0.93, 3.88]	0.50 [−0.04, 0.90]	0.20	0.133
	Absolute sagittal lip step	0.51 [0.19, 1.02]	0.57 [0.27, 1.35]	−0.12 [−0.22, 0.03]	−0.16	0.248
	3D commissure distance	1.95 [0.94, 3.90]	2.00 [0.83, 3.51]	0.14 [−0.34, 0.57]	0.13	0.297

Positive CONV − PHON differences and positive rank–biserial r values indicate lower absolute error under PHON. Confidence intervals (CI) are percentile-bootstrap intervals for the participant-level median paired difference (10,000 resamples). *p*-values are two-sided Wilcoxon signed-rank tests adjusted by Holm within the nine-measure SMILE family and six-measure REST family. IQR, interquartile range.

**Table 3:** State-by-protocol contrasts for the six measures shared by SMILE and REST (*n* = 162).

Measurement	SMILE Effect, mm	REST Effect, mm	State-by-Protocol Contrast [95% CI], mm	Rank–Biserial r	Holm-Adjusted *p*
3D intercanthal distance	−0.16	0.20	−0.47 [−0.70, −0.25]	−0.34	<0.001
3D nasal width	−0.13	0.08	0.17 [−0.12, 0.51]	−0.07	0.442
3D interlabial distance	0.83	−0.20	1.53 [0.86, 2.38]	0.46	<0.001
Absolute sagittal Sn-Li separation	0.16	0.50	−0.44 [−1.07, 0.38]	−0.12	0.382
Absolute sagittal lip step	0.47	−0.12	0.68 [0.35, 0.95]	0.46	<0.001
3D commissure distance	−0.45	0.14	−0.53 [−1.35, 0.22]	−0.25	0.020

Protocol effect = CONV error − PHON error. State-by-protocol contrast = SMILE protocol effect − REST protocol effect. Positive contrasts indicate a larger PHON-related reduction in error during SMILE. Confidence intervals summarize the median contrast; *p*-values are Wilcoxon signed-rank tests with Holm correction across six shared measures.

## Data Availability

Original 3D facial images and raw XYZ landmark coordinates are not publicly available because they constitute potentially identifiable biometric data and participant consent did not permit unrestricted release. Requests for controlled access may be directed to the corresponding authors and will require institutional ethics approval and a data-use agreement.

## Supplementary Materials

The following supporting information can be downloaded at https://www.mdpi.com/article/10.3390/bioengineering13090994/s1; Table S1, Operational instructions for conventional and phoneme-guided tasks; Table S2, Facial landmarks and anatomical definitions; Table S3, Derived 3D measures and state applicability; Table S4, State-specific regional landmark-displacement comparisons; Table S5, Within-protocol TEM, ICC(2,1), and Bland–Altman agreement; Table S6, Secondary stable-landmark acquisition-quality indicators in the complete cohort; Table S7, Complete descriptive summary of absolute R1–R2 errors in the full cohort; Figure S1, Selected state-specific absolute-error distributions in the complete cohort; Figure S2, Empirical cumulative distributions of the stable-landmark displacement index; Figure S3, Bland–Altman agreement for 3D interlabial distance by facial state and acquisition instruction; Figure S4, Empirical cumulative distributions of absolute commissure-distance error during SMILE and REST.

## Abbreviations

3D, three-dimensional; CI, confidence interval; CONV, conventional acquisition instruction; GRRAS, Guidelines for Reporting Reliability and Agreement Studies; ICC, intraclass correlation coefficient; ICP, iterative closest point; IQR, interquartile range; PHON, phoneme-guided acquisition instruction; P90, 90th percentile; R1 and R2, first and second repeated acquisitions; TEM, technical error of measurement.
