# Supplementary material for: Repeatability of Phoneme-Guided Versus Conventional Acquisition of Smiling and Rest-Related Facial States Using 3D Stereophotogrammetry: A Within-Participant Study
Source: Bioengineering (Basel). 2026 Aug 27;13(9):994. doi: 10.3390/bioengineering13090994 (PMC13603687; doi:10.3390/bioengineering13090994)
Supplement: Supplementary file 1 [file bioengineering-13-00994-s001.zip › bioengineering-4499448-supplementary.pdf]

bioengineering

Supplementary

Repeatability of Phoneme-Guided Versus Conventional Acquisition of Smiling and Rest-Related Facial States Using 3D Stereophotogrammetry: A Within-Participant Study

Weijing Dang [1,2,3,4,†], Liang Lyu [1,2,3,5,†,*], Yong Shen [2,4], Ji Wang [1,2,3,4], Yi Yu [1,2,3], Qirui Wang [1,2,3], Mingjin Zhang [1,2,3], Jiajia Zheng [2,3,5] and Dawei Liu [1,2,3,*]

[1] Department of Orthodontics, Peking University School and Hospital of Stomatology and National Center of Stomatology, Beijing 100081, China
[2] National Clinical Research Center for Oral Diseases and National Engineering Research Center of Oral Biomaterials and Digital Medical Devices, Beijing 100081, China
[3] Beijing Key Laboratory of Digital Stomatology, Beijing 100081, China
[4] Department of Nursing, Peking University School and Hospital of Stomatology and National Center of Stomatology, Beijing 100081, China
[5] Eighth Clinical Division (Shijingshan Clinical Division), Peking University School and Hospital of Stomatology and National Center of Stomatology, Beijing 100043, China
* Correspondence: lvliang_1996@126.com (L.L.); liudawei@bjmu.edu.cn (D.L.)
† These authors contributed equally to this work.

Table S1. Operational instructions for conventional and phoneme-guided tasks.

State	Protocol	Instruction	Motor target	Replicates	Interpretation
SMILE	CONV	"Please smile naturally and hold still."	Participant-defined posed smile.	Two consecutive accepted acquisitions (R1 then R2).	Conventional nonverbal instruction.
SMILE	PHON	"Please say 'ch' once; after the sound ends, keep the resulting mouth and lip position and hold still until capture."	Explicit speech-related perioral configuration.	Two consecutive accepted acquisitions (R1 then R2).	Not assumed to be interchangeable with a natural posed smile.
REST	CONV	"Please relax your facial muscles, close your lips naturally, and hold still."	Participant-defined relaxed rest.	Two consecutive accepted acquisitions (R1 then R2).	Conventional nonverbal instruction.
REST	PHON	"Please make a gentle 'mmm' sound and maintain the resulting closed-lip position until capture."	Explicit bilabial closed-lip reference configuration.	Two consecutive accepted acquisitions (R1 then R2).	Not assumed to be identical to unconstrained physiological rest.

Table S2. Facial landmarks and anatomical definitions.

Landmark	Full name	Anatomical definition	Region	Included in stable-landmark index
EnR	Right endocanthion	Medial commissure of the right palpebral fissure.	Upper face	Yes
EnL	Left endocanthion	Medial commissure of the left palpebral fissure.	Upper face	Yes
ExR	Right exocanthion	Lateral commissure of the right palpebral fissure.	Upper face	Yes
ExL	Left exocanthion	Lateral commissure of the left palpebral fissure.	Upper face	Yes
Prn	Pronasale	Most anterior point of the nasal tip.	Midface	Yes

AlR	Right alare	Most lateral point on the contour of the right nasal ala.	Midface	Yes
AlL	Left alare	Most lateral point on the contour of the left nasal ala.	Midface	Yes
Sn	Subnasale	Midline point at the junction of the columella base and upper lip.	Midface	No
CphR	Right crista philtri	Right peak of Cupid's bow at the philtral column/vermilion junction.	Lower face	No
CphL	Left crista philtri	Left peak of Cupid's bow at the philtral column/vermilion junction.	Lower face	No
Stms	Stomion superius	Midline point on the lower margin of the upper lip at the oral fissure.	Lower face	No
Stmi	Stomion inferius	Midline point on the upper margin of the lower lip at the oral fissure.	Lower face	No
Ls	Labrale superius	Most anterior midline point on the upper vermilion border.	Lower face	No
Li	Labrale inferius	Most anterior midline point on the lower vermilion border.	Lower face	No
ChR	Right cheilion	Right labial commissure where the vermilion borders meet.	Lower face	No
ChL	Left cheilion	Left labial commissure where the vermilion borders meet.	Lower face	No
Gn	Soft-tissue gnathion	Most inferior/anterior-inferior midline point on the soft-tissue chin.	Lower face	No
Pog	Soft-tissue pogonion	Most anterior midline point on the soft-tissue chin.	Lower face	No
GoR	Right soft-tissue gonion	Soft-tissue point over the right mandibular angle.	Lower face	No
GoL	Left soft-tissue gonion	Soft-tissue point over the left mandibular angle.	Lower face	No

Table S3. Derived 3D measures and state applicability.

Measurement	Definition	Measurement family	Calculation	States
3D intercanthal distance	Euclidean distance EnR-EnL	Control and nasal	3D Euclidean distance	SMILE, REST
3D nasal width	Euclidean distance AlR-AlL	Control and nasal	3D Euclidean distance	SMILE, REST
3D Sn-Gn distance	Euclidean distance Sn-Gn	Lower-face 3D distance	3D Euclidean distance	SMILE
3D Stmi-Gn distance	Euclidean distance Stmi-Gn	Lower-face 3D distance	3D Euclidean distance	SMILE
3D interlabial distance	Euclidean distance Stms-Stmi	Lower-face 3D distance	3D Euclidean distance	SMILE, REST
Absolute sagittal Sn-Li separation	Absolute Z-axis difference $\lvert Z_{Sn} - Z_{Li} \rvert$	Sagittal component	Absolute sagittal (Z-axis) difference	SMILE, REST
Absolute sagittal lip step	Absolute Z-axis difference $\lvert Z_{Ls} - Z_{Li} \rvert$	Sagittal component	Absolute sagittal (Z-axis) difference	SMILE, REST
3D commissure distance	Euclidean distance ChR-ChL	Transverse 3D distance	3D Euclidean distance	SMILE, REST
3D bigonial distance	Euclidean distance GoR-GoL	Transverse 3D distance	3D Euclidean distance	SMILE

Table S4. State-specific regional landmark-displacement comparisons.

State	Region	CONV median [IQR], mm	PHON median [IQR], mm	CONV − PHON median difference [95% CI], mm	Rank–biserial r	Raw p	Adjusted p
SMILE	Upper face	1.93 [1.46, 2.79]	1.98 [1.48, 2.59]	−0.06 [−0.18, 0.11]	0.00	0.975	0.975
SMILE	Midface	3.65 [2.60, 4.57]	3.41 [2.57, 4.77]	−0.02 [−0.29, 0.43]	0.08	0.401	0.801
SMILE	Lower face	4.70 [3.73, 6.44]	4.22 [3.41, 5.43]	0.45 [0.03, 0.76]	0.20	0.024	0.072
REST	Upper face	1.90 [1.44, 3.07]	1.85 [1.45, 2.79]	0.14 [0.01, 0.32]	0.26	0.005	0.014
REST	Midface	3.77 [2.67, 6.12]	3.77 [2.87, 5.66]	0.23 [−0.12, 0.71]	0.23	0.010	0.014
REST	Lower face	3.74 [3.20, 5.12]	3.74 [3.19, 5.01]	0.24 [−0.02, 0.48]	0.25	0.006	0.014

P-values are two-sided paired Wilcoxon signed-rank tests; adjusted p-values use Holm correction across the three regions within each state.

Table S5. Within-protocol TEM, ICC(2,1), and Bland–Altman agreement.

State	Measurement	Protocol	*n*	TEM, mm	ICC(2,1) [95% CI]	Bias, mm	95% limits of agreement, mm
SMILE	3D intercanthal distance	CONV	162	0.93	0.892 [0.856, 0.920]	−0.16	−2.72 to 2.40
SMILE	3D intercanthal distance	PHON	162	1.18	0.828 [0.773, 0.871]	−0.14	−3.42 to 3.14
SMILE	3D nasal width	CONV	162	1.32	0.855 [0.808, 0.892]	−0.40	−4.00 to 3.19
SMILE	3D nasal width	PHON	162	1.96	0.692 [0.605, 0.764]	−0.86	−6.05 to 4.33
SMILE	3D Sn-Gn distance	CONV	162	5.28	0.669 [0.579, 0.743]	−3.66	−16.45 to 9.14
SMILE	3D Sn-Gn distance	PHON	162	2.84	0.762 [0.690, 0.819]	−1.00	−8.65 to 6.64
SMILE	3D Stmi-Gn distance	CONV	162	2.05	0.730 [0.650, 0.794]	0.69	−4.84 to 6.22
SMILE	3D Stmi-Gn distance	PHON	162	1.69	0.803 [0.741, 0.852]	0.20	−4.47 to 4.88
SMILE	3D interlabial distance	CONV	162	5.95	0.444 [0.332, 0.547]	−4.91	−18.33 to 8.52
SMILE	3D interlabial distance	PHON	162	3.56	0.339 [0.209, 0.460]	−2.04	−11.09 to 7.01
SMILE	Absolute sagittal Sn-Li separation	CONV	162	2.97	0.699 [0.613, 0.769]	−1.33	−9.16 to 6.49
SMILE	Absolute sagittal Sn-Li separation	PHON	162	3.20	0.596 [0.489, 0.686]	−1.02	−9.69 to 7.64
SMILE	Absolute sagittal lip step	CONV	162	2.63	0.564 [0.457, 0.656]	−1.66	−8.19 to 4.87
SMILE	Absolute sagittal lip step	PHON	162	1.29	0.762 [0.690, 0.819]	−0.54	−3.96 to 2.89
SMILE	3D commissure distance	CONV	162	2.31	0.831 [0.777, 0.873]	−0.54	−6.87 to 5.80
SMILE	3D commissure distance	PHON	162	5.46	0.309 [0.175, 0.434]	−2.86	−16.95 to 11.23
SMILE	3D bigonial distance	CONV	162	6.20	0.687 [0.597, 0.760]	1.95	−14.87 to 18.76
SMILE	3D bigonial distance	PHON	162	3.94	0.848 [0.799, 0.887]	−0.37	−11.30 to 10.56
REST	3D intercanthal distance	CONV	162	1.81	0.574 [0.461, 0.669]	0.17	−4.86 to 5.19
REST	3D intercanthal distance	PHON	162	1.03	0.860 [0.814, 0.896]	−0.02	−2.89 to 2.85
REST	3D nasal width	CONV	162	2.42	0.563 [0.451, 0.659]	0.85	−5.66 to 7.36
REST	3D nasal width	PHON	162	1.49	0.799 [0.736, 0.849]	−0.08	−4.21 to 4.06
REST	3D interlabial distance	CONV	162	6.24	0.004 [−0.146, 0.155]	1.47	−15.64 to 18.58
REST	3D interlabial distance	PHON	162	4.42	0.089 [−0.049, 0.227]	2.16	−9.39 to 13.70
REST	Absolute sagittal Sn-Li separation	CONV	162	3.20	0.531 [0.418, 0.630]	1.75	−6.46 to 9.96
REST	Absolute sagittal Sn-Li separation	PHON	162	2.79	0.613 [0.508, 0.700]	0.96	−6.56 to 8.47
REST	Absolute sagittal lip step	CONV	162	1.44	0.498 [0.375, 0.604]	0.50	−3.39 to 4.40

REST	Absolute sagittal lip step	PHON	162	1.70	0.570 [0.460, 0.664]	0.77	−3.73 to 5.26
REST	3D commissure distance	CONV	162	4.89	0.384 [0.255, 0.501]	2.70	−9.81 to 15.21
REST	3D commissure distance	PHON	162	2.63	0.662 [0.566, 0.740]	−0.28	−7.58 to 7.02

TEM, technical error of measurement; ICC, intraclass correlation coefficient; CI, confidence interval. ICC(2,1) denotes a two-way random-effects, absolute-agreement, single-measure model.

Table S6. Secondary stable-landmark acquisition-quality indicators in the complete cohort (*n* = 162).

State	*n*	Upper fence, mm	CONV median [IQR], mm	PHON median [IQR], mm	CONV – PHON median difference [95% CI], mm	Rank–biserial r	Holm p, continuous index	High-displacement flags, *n* (CONV; PHON)	Holm p, exact McNemar
SMILE	162	4.63	2.16 [1.59, 2.87]	2.16 [1.69, 2.81]	−0.05 [−0.28, 0.19]	0.01	0.882	14; 10	0.481
REST	162	5.63	1.91 [1.48, 3.07]	2.09 [1.52, 3.26]	0.21 [−0.07, 0.31]	0.20	0.051	34; 27	0.031

The stable-landmark displacement index is the median R1–R2 Euclidean displacement across EnR, EnL, ExR, ExL, Prn, AlR, and AlL. The state-specific upper fence was calculated as Q3 + 1.5 × IQR from the pooled protocol-level distribution and was used descriptively only; no observation was excluded. Continuous p-values are from paired Wilcoxon tests and flag p-values from exact McNemar tests, with Holm correction across SMILE and REST.

Table S7. Complete descriptive summary of absolute R1–R2 errors in the full cohort (*n* = 162).

State	Measurement	*n*	CONV median [IQR], mm	CONV mean, mm	CONV P90, mm	PHON median [IQR], mm	PHON mean, mm	PHON P90, mm
SMILE	3D intercanthal distance	162	0.76 [0.31, 1.37]	0.99	2.18	0.96 [0.45, 1.75]	1.26	2.90
SMILE	3D nasal width	162	1.37 [0.55, 2.12]	1.51	3.29	1.36 [0.69, 2.35]	1.97	4.52
SMILE	3D Sn-Gn distance	162	3.79 [1.34, 6.52]	5.30	13.88	2.36 [1.03, 4.06]	3.01	6.73
SMILE	3D Stmi-Gn distance	162	1.56 [0.82, 2.89]	2.16	4.69	1.38 [0.70, 2.42]	1.78	3.51
SMILE	3D interlabial distance	162	3.48 [1.29, 9.41]	6.01	15.15	1.84 [0.71, 4.02]	3.25	8.96
SMILE	Absolute sagittal Sn-Li separation	162	2.25 [0.97, 4.42]	3.09	6.75	2.16 [1.01, 4.84]	3.24	7.37
SMILE	Absolute sagittal lip step	162	1.33 [0.41, 3.54]	2.44	5.36	0.66 [0.30, 1.70]	1.20	2.88
SMILE	3D commissure distance	162	1.50 [0.68, 3.02]	2.28	5.27	1.89 [0.92, 4.15]	4.27	11.92
SMILE	3D bigonial distance	162	3.70 [1.68, 6.81]	5.07	9.45	3.69 [1.80, 6.19]	4.43	9.22
REST	3D intercanthal distance	162	1.12 [0.52, 2.10]	1.69	3.48	0.85 [0.34, 1.55]	1.11	2.56
REST	3D nasal width	162	1.29 [0.60, 2.75]	2.16	4.79	1.41 [0.55, 2.44]	1.64	3.08
REST	3D interlabial distance	162	1.21 [0.46, 2.72]	3.46	9.62	1.37 [0.64, 3.06]	3.36	9.69
REST	Absolute sagittal Sn-Li separation	162	2.71 [1.18, 4.33]	3.39	7.44	1.99 [0.93, 3.88]	2.86	6.69
REST	Absolute sagittal lip step	162	0.51 [0.19, 1.02]	1.13	3.51	0.57 [0.27, 1.35]	1.31	3.15
REST	3D commissure distance	162	1.95 [0.94, 3.90]	4.10	12.23	2.00 [0.83, 3.51]	2.66	5.86

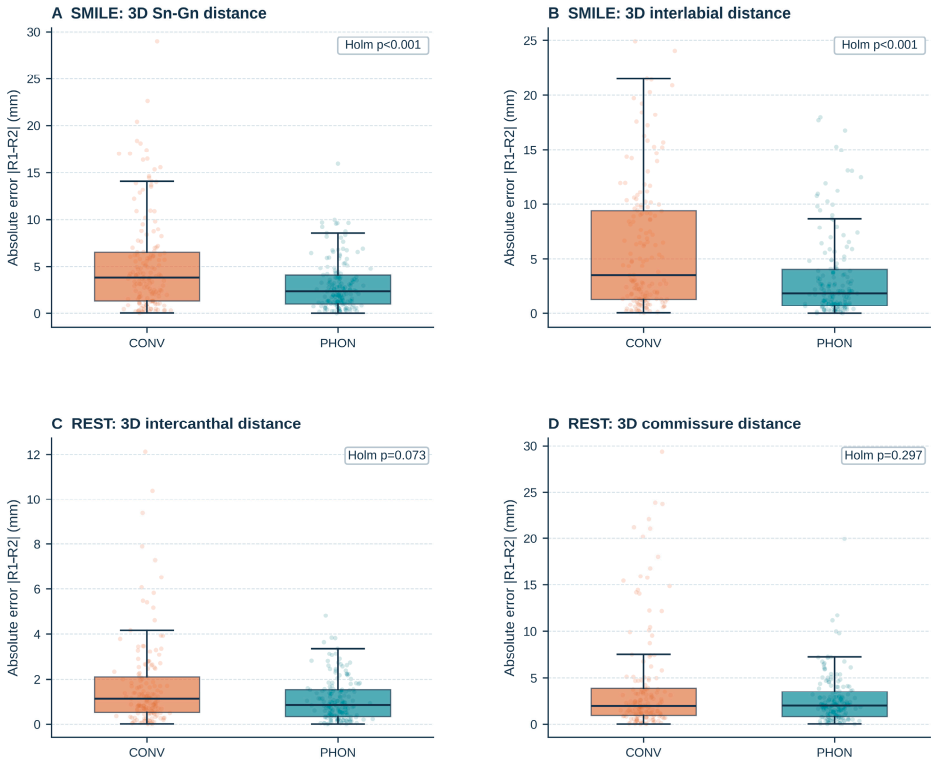

Figure S1. Selected state-specific absolute-error distributions in the complete cohort. Boxes show the interquartile range and median; individual participant observations are overlaid. P-values are Holm-adjusted within the corresponding facial-state family.

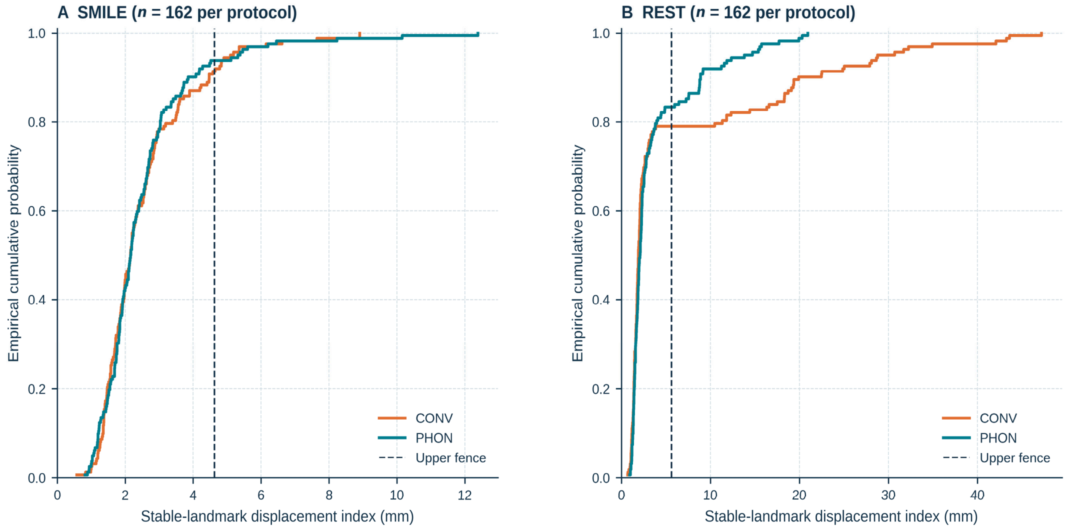

Figure S2. Empirical cumulative distributions of the stable-landmark displacement index under CONV and PHON during SMILE and REST (n = 162 per protocol). Dashed vertical lines show the state-specific pooled upper fences. The continuous index and threshold flag were exploratory acquisition-quality indicators and did not determine inclusion.

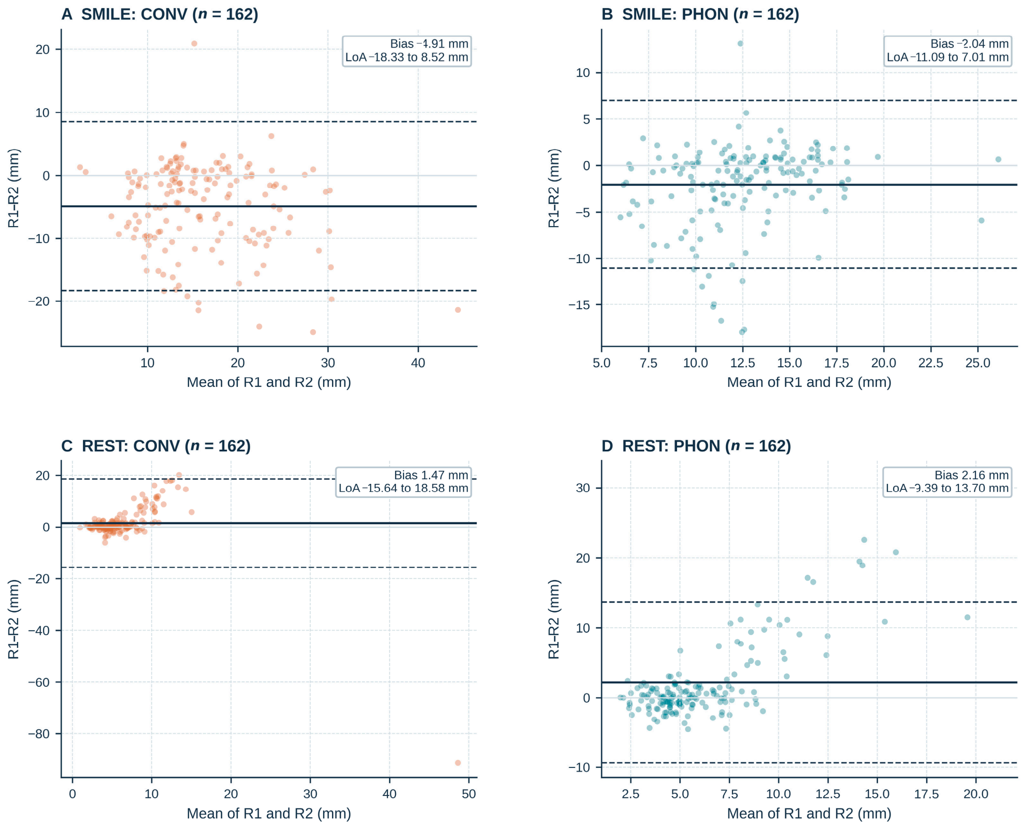

Figure S3. Bland–Altman agreement for 3D interlabial distance by facial state and acquisition instruction. Solid lines show mean R1–R2 bias; dashed lines show the 95% limits of agreement.

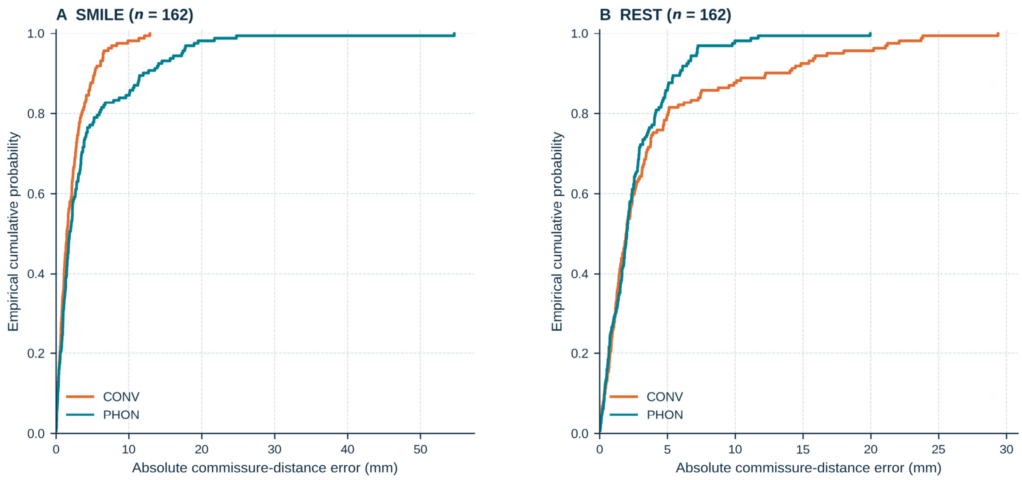

Figure S4. Empirical cumulative distributions of absolute commissure-distance error during SMILE and REST. Rightward displacement indicates a larger proportion of high-error observations.
